# Decreased fracture incidence with traditional Chinese medicine therapy in patients with osteoporosis: a nationwide population-based cohort study

**DOI:** 10.1186/s12906-019-2446-3

**Published:** 2019-02-04

**Authors:** Yu-Chi Wang, Jen-Huai Chiang, Hsin-Cheng Hsu, Chun-Hao Tsai

**Affiliations:** 1 Department of Chinese Medicine, China Medical University Hospital, Taichung, Taiwan; 2 Management Office for Health Data, China Medical University Hospital, Taichung, Taiwan; 3 College of Medicine, China Medical University, Taichung, Taiwan; 4 Graduate Institute of Integrated Medicine, College of Chinese Medicine, Research Center for Chinese Medicine and Acupuncture, China Medical University, Taichung, Taiwan; 5 College of Post-baccalaureate Chinese Medicine, China Medical University, Taichung, Taiwan; 6 Department of Orthopedics, China Medical University Hospital, Taichung, Taiwan; 7 School of Medicine, China Medical University, Taichung, Taiwan; 8 School of Medicine and Department of Orthopedics, China Medical University, China Medical University Hospital, No.91 Hsueh-Shih Road, Taichung, Taiwan

**Keywords:** National Health Insurance Research Database, Osteoporotic fracture, Traditional Chinese medicine

## Abstract

**Background:**

There are no published studies regarding the efficacy of traditional Chinese medicine (TCM) for the prevention of osteoporotic fracture. Therefore, we conducted this nationwide, population-based cohort study to investigate the probable effect of TCM to decrease the fracture rate.

**Methods:**

We identified cases with osteoporosis and selected a comparison group that was frequency-matched according to sex, age (per 5 years), diagnosis year of osteoporosis, and index year. The difference between the two groups in the development of fracture was estimated using the Kaplan–Meier method and the log-rank test.

**Results:**

After inserting age, gender, urbanization level, and comorbidities into the Cox’s proportional hazard model, patients who used TCM had a lower hazard ratio (HR) of fracture (adjusted HR: 0.47, 95% CI: 0.37–0.59) compared to the non-TCM user group. The Kaplan-Meier curves showed that osteoporosis patients who used TCM had a lower incidence of fracture events than those who did not (*p* < 0.00001). Our study also demonstrated that the longer the TCM use, the lesser the fracture rate.

**Conclusion:**

Our study showed that TCM might have a positive impact on the prevention of osteoporotic fracture.

## Background

Osteoporosis is defined as a skeletal disorder that occurs with the decrease in bone density and quality, leading to an increased risk of fracture [1]. The most frequent fracture areas are the hip, wrist, and spine. In the United States, 1.5 million osteoporotic patients over 50 years of age suffer from hip fractures each year [2] and over 3.5 million fragility fractures occur each year in Europe [3]. Osteoporotic fracture is an economic burden which cost US$17 billion annually in the United States in 2005 and €37 billion in Europe in 2010 [3, 4]. In Taiwan, the incidence of hip fracture increases 9.3% annually, with 13,892 women and 8616 men over 50 years of age suffering from hip fracture in 2010. According to the data from Health Promotion Administration, approximately 40% of women over the age of 70 need long-term bed rest and care due to hip fracture, and 10% die from hip fracture in Taiwan. In Taiwan, every case requires more than NT$100,000 during the acute phase, and more resources are needed in the long run [5]. The incidence of fractures is expected to increase over the next 30 years because of the increase in the aged population [6]. Half of all hip fractures will occur in Asia by 2050 where the amount of older people will be most markedly increased [7].

There are numerous drugs available for treating osteoporosis; however, only bisphosphonates and denosumab have been demonstrated to have antifracture efficacy. Besides, only teriparatide and intact Parathyroid hormone (PTH) are approved to stimulate bone formation; the others are antiresorptive agents. In view of this, people might wonder whether other therapies have been ignored. There are no published studies regarding the efficacy of traditional Chinese medicine (TCM) for the prevention of osteoporotic fracture. Some studies revealed the effects of TCM, such as *Cistanche deserticola* extract, Baicalin, Semen Astragali Complanati decoction, and Rhizoma Cibotii decoction, in regard to promoting bone formation, mineralization, and decreasing bone loss [8–10]. However, these studies used cell-lines and animal models. Therefore, large-scale, population-based analyses examining the preventative effect of TCM herbal products for osteoporotic fracture are needed.

To investigate the probable effect of TCM to decrease the fracture rate in patients with osteoporosis, we analyzed the National Health Insurance Research Database (NHIRD) of Taiwan from 2000 to 2010. TCM has been reimbursed by the National Health Insurance (NHI) program since 1996 in Taiwan, including Chinese herbal products, acupuncture/moxibustion, and manipulative therapy [11]. At the end of 2011, more than 99% of the population were enrolled in the NHI program [12]. This study provides important information for clinicians and shows that Chinese herbal prescriptions could also be useful for further pharmacological investigation or clinical trials.

## Methods

### Data source

Taiwan launched the mandatory National Health Insurance (NHI) program in 1995 and has been reimbursing Western and TCM since 1996. TCM treatment includes Chinese herbal medicine, acupuncture, and moxibustion therapy in ambulatory clinics. Large computerized data (NHIRD) was used to perform our nationwide population-based cohort study. We used the LHID2000 (Longitudinal Health Insurance Database 2000) provided by the National Health Insurance Administration, which is managed by the National Health Research Institutes. The LHID2000 includes data from 1 million randomly selected patients who were NHI beneficiaries in 2000. Similar distributions of beneficiaries based on age and gender of beneficiary age and gender in the LHID2000 and the general NHI database were observed. The registration and claim dataset from the LHID2000 spans the years 2000 to 2011. Ambulatory care claims contain an individual’s gender, date of birth, visit date, and the International Classification of Disease, Ninth Revision, Clinical Modification (ICD-9-CM) codes for three primary diagnoses. Inpatient claims contain ICD-9-CM codes for the principal diagnosis and up to four secondary diagnoses. A disease diagnosis without valid supporting clinical findings may be considered as medical fraud by the NHI with a penalty of 100-fold the payment claimed by the treating physician or hospital. This study was approved by the Institutional Review Board of China Medical University (CMUH104-REC2–115).

### Study population

**Fig. 1:**
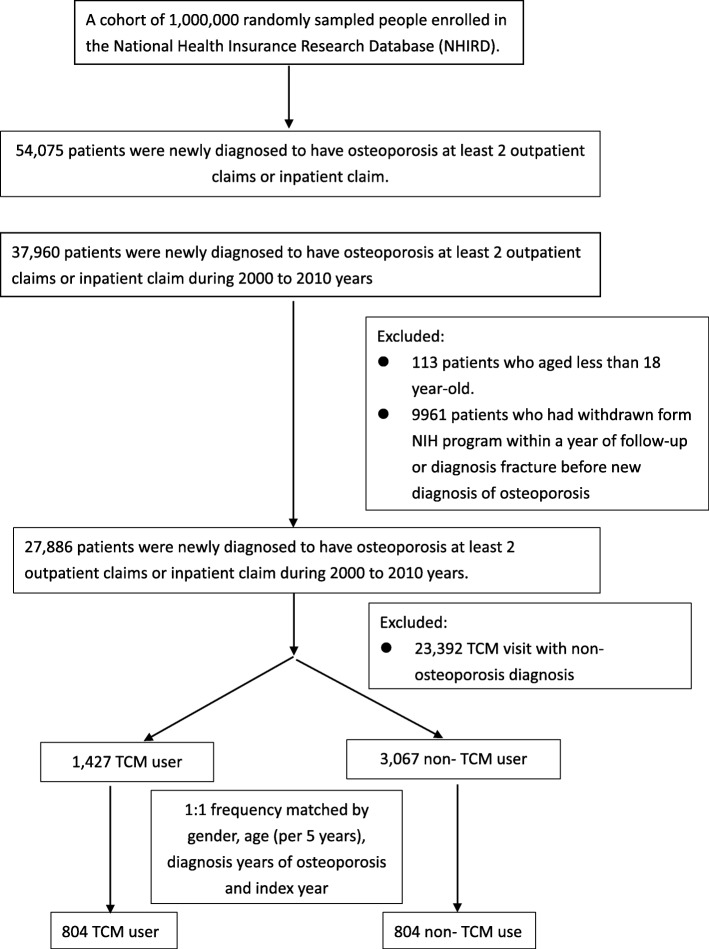
The recruitment flowchart of subjects from the one million samples randomly selected from the National Health Insurance Research Database (NHIRD) in Taiwan. There were a total of 54,075 osteoporosis patients registered in the NHIRD, with 37,960 patients diagnosed between 2000 and 2010. After ruling out patients with missing information and aged > 18 years, as well as matching 1:1 by sex, age, diagnosis year of osteoporosis, and index year, both groups contained 804 patients

Our population cohort study used newly diagnosed osteoporosis patients (aged ≥18 years) identified between 2000 and 2010 and followed up until the December 31, 2011 or the first manifestation of fracture. Subjects with osteoporosis were required to have at least two outpatient claims or at least one inpatient claim with the diagnosis of ICD—CM code 733.0 during the study period. The exclusion criteria included less than 18 years old, incomplete information of age and sex, and withdrawal from the NHIRD during the follow-up period. Patients who received TCM treatment for their osteoporosis from the initial diagnosis to December 31, 2010, were identified as the TCM user cohort. The date of the first TCM treatment after a new diagnosis of osteoporosis was used as the index date for the cohort group. No diagnosis and TCM treatment code in the database was categorized as non-TCM users. Figure 1 shows the subject recruitment flowchart of osteoporosis patients from the NHIRD in Taiwan.

### Covariate assessment

Sociodemographic factors included age and sex. Patients were divided into two groups based on their age; < 65 years and ≥ 65 years old. The townships in which subjects registered for insurance were grouped into four levels of urbanization based on a score calculated by incorporating variables indicating population density (people/km^2^), population ratio of different educational levels, population ratio of elderly, population ratio of agriculture workers, and the number of physicians per 100,000 people [13]. Baseline comorbidities comprised alcohol-related disease (ICD-9-CM: 291, 303, 305.00, 305.01, 305.02, 305.03, 571.0–571.3, 790.3, V11.3), cancer (140–208), cardiovascular disease (410–414, 428, 430–438, 440–448), chronic kidney disease (585–586, 588.8–588.9), chronic obstructive pulmonary disease (491, 492, 493, 496), diabetes mellitus (250), dementia (290.0, 290.1, 290.2, 290.3, 290.4, 294.1, 331.0), depression (296.2, 296.3, 300.4, 311), hyperlipidemia (272.0, 272.1, 272.2, 272.3, 272.4), hypertension (401–405), and Parkinson’s disease (332.x).

### Data analysis

Categorical variables were reported as numbers and percentages. The difference in proportions was assessed using the chi-square test. Cox’s proportional hazard model estimated hazard ratios (HR) of TCM usage on fractures. The difference in fracture development between the two groups was estimated using the Kaplan–Meier method and the log-rank test. Statistical analysis was performed and figures were created using SAS 9.4 (SAS Institute, Cary, NC, U.S.A.) and R software, with *p* < 0.05 in two-tailed tests indicating statistical significance.

## Results

Overall, there were 1427 TCM users and 3067 non-TCM users among the osteoporosis patients. After frequency matching, there were 804 patients in the TCM user and non-TCM user groups. Table 1 shows the baseline characteristics according to TCM usage. Osteoporosis patients in both groups had a similar distribution of gender and age. The mean age of the TCM user and non-TCM user groups was 64.48 ± 11.08 and 64.57 ± 11.08, respectively, and the female and male percentages were 76.49 and 23.51%, respectively. Compared with non-TCM user group, TCM users had a higher proportion of chronic obstructive pulmonary disease and hyperlipidemia, but had a lower proportion of cancer.

**Table 1:** Characteristics of osteoporosis patients according to use of traditional Chinese medicine

Variable	TCM				
	No (*N* = 804)		Yes (*N* = 804)		*p*-value
	n	%	n	%	
Gender					0.99*
Female	615	76.49	615	76.49	
Male	189	23.51	189	23.51	
Age group, year					0.99*
< 65	386	48.01	386	48.01	
≥ 65	418	51.99	418	51.99	
Mean (SD)	64.57 (11.08)		64.48 (11.08)		0.8813^a^
Urbanization level^†^					0.0104*
1 (highest)	207	25.75	240	29.85	
2	238	29.6	226	28.11	
3	106	13.18	134	16.67	
4 (lowest)	253	31.47	204	25.37	
Baseline comorbidity					
Alcohol-related disease	4	0.5	1	0.12	0.3742^b^
Cancer	45	5.6	22	2.74	0.0041*
Cardiovascular disease	307	38.18	305	37.94	0.9182*
Chronic kidney disease	25	3.11	25	3.11	0.99*
Chronic obstructive pulmonary disease	188	23.38	241	29.98	0.0028*
Diabetes mellitus	212	26.37	185	23.01	0.1184*
Dementia	19	2.36	12	1.49	0.2043*
Depression	44	5.47	54	6.72	0.2972*
Hyperlipidemia	168	20.9	218	27.11	0.0035*
Hypertension	447	55.6	416	51.74	0.1211*
Parkinson’s disease	8	1	11	1.37	0.4887*
Interval between diagnosis and initial TCM use, mean (days)			611		
Follow-up time, mean (median; years)	3.75 (2.86)		5.38 (5.18)		

*Chi-Square Test, ^a^ t-test, ^b^Fisher’s exact test

^†^: The urbanization level was categorized into four levels based on the population density of the residential area, with level 1 as the most urbanized and level 4 as the least urbanized

Traditional Chinese medicine (TCM) included Chinese herbal remedies, acupuncture, and manipulative

A total of 323 patients were newly diagnosed with a fracture during the follow-up period (59% non-TCM users and 40% TCM users). A reduced risk of fracture recurrence was associated with TCM use (Crude HR: 0.50, 95% CI: 0.4–0.63). After inserting age, gender, urbanization level, alcohol-related disease, cancer, cardiovascular disease, chronic kidney disease, chronic obstructive pulmonary disease, diabetes mellitus, dementia, depression, hyperlipidemia, hypertension, and Parkinson’s disease into the Cox’s proportional hazard model, TCM use had a lower HR of fracture (adjusted HR: 0.47, 95% CI: 0.37–0.59) compared to the non-TCM user group (Table 2).

**Table 2:** Cox model with hazard ratios and 95% confidence intervals of fracture associated with TCM and covariates among osteoporosis patients

Variable	Fracture no. (*n* = 323)	Crude^*^			Adjusted^†^		
		HR	(95%CI)	p-value	HR	(95%CI)	*p*-value
TCM use							
No	193	1.00	reference		1.00	reference	
Yes	130	0.50	(0.4–0.63)	<.0001	0.47	(0.37–0.59)	<.0001
Gender							
Female	266	1.00	reference		1.00	reference	
Male	57	0.78	(0.59–1.04)	0.0939	0.58	(0.43–0.79)	0.0004
Age group, year							
< 65	107	1.00	reference		1.00	reference	
≥ 65	216	2.28	(1.81–2.87)	<.0001	2.62	(2.03–3.39)	<.0001
Urbanization level							
1 (highest)	73	1.00	reference		1.00	reference	
2	94	1.28	(0.94–1.73)	0.1173	1.24	(0.91–1.69)	0.1665
3	52	1.46	(1.02–2.08)	0.0372	1.43	(0.99–2.05)	0.0534
4 (lowest)	104	1.54	(1.14–2.07)	0.0049	1.31	(0.97–1.77)	0.0824
Baseline comorbidity							
Alcohol-related disease (Yes vs. No)	4	5.20	(1.94–13.96)	0.0011	4.38	(1.6–12.02)	0.0041
Cancer (Yes vs. No)	9	0.83	(0.43–1.61)	0.5758	0.70	(0.36–1.37)	0.2979
Cardiovascular disease (Yes vs. No)	139	1.38	(1.1–1.71)	0.0046	1.07	(0.83–1.38)	0.5856
Chronic kidney disease (Yes vs. No)	9	1.22	(0.63–2.36)	0.5608	1.05	(0.53–2.07)	0.8909
Chronic obstructive pulmonary (Yes vs. No)disease	92	1.25	(0.98–1.59)	0.0759	1.20	(0.93–1.54)	0.1688
Diabetes mellitus (Yes vs. No)	87	1.20	(0.94–1.54)	0.1442	1.11	(0.85–1.44)	0.4518
Dementia (Yes vs. No)	6	1.62	(0.72–3.64)	0.2432	1.00	(0.43–2.29)	0.9909
Depression (Yes vs. No)	25	1.41	(0.94–2.12)	0.0972	1.62	(1.05–2.5)	0.0285
Hyperlipidemia (Yes vs. No)	62	0.79	(0.6–1.04)	0.0922	0.73	(0.54–0.98)	0.0352
Hypertension (Yes vs. No)	186	1.28	(1.03–1.59)	0.0292	0.92	(0.72–1.19)	0.5362
Parkinson’s disease (Yes vs. No)	5	1.25	(0.52–3.02)	0.6248	0.92	(0.37–2.27)	0.8498

Crude HR^*^ represented relative hazard ratio; Adjusted HR^†^ represented adjusted hazard ratio: mutually adjusted for TCM use, age, gender, urbanization level and baseline comorbidity in Cox proportional hazard regression

**Fig. 2:**
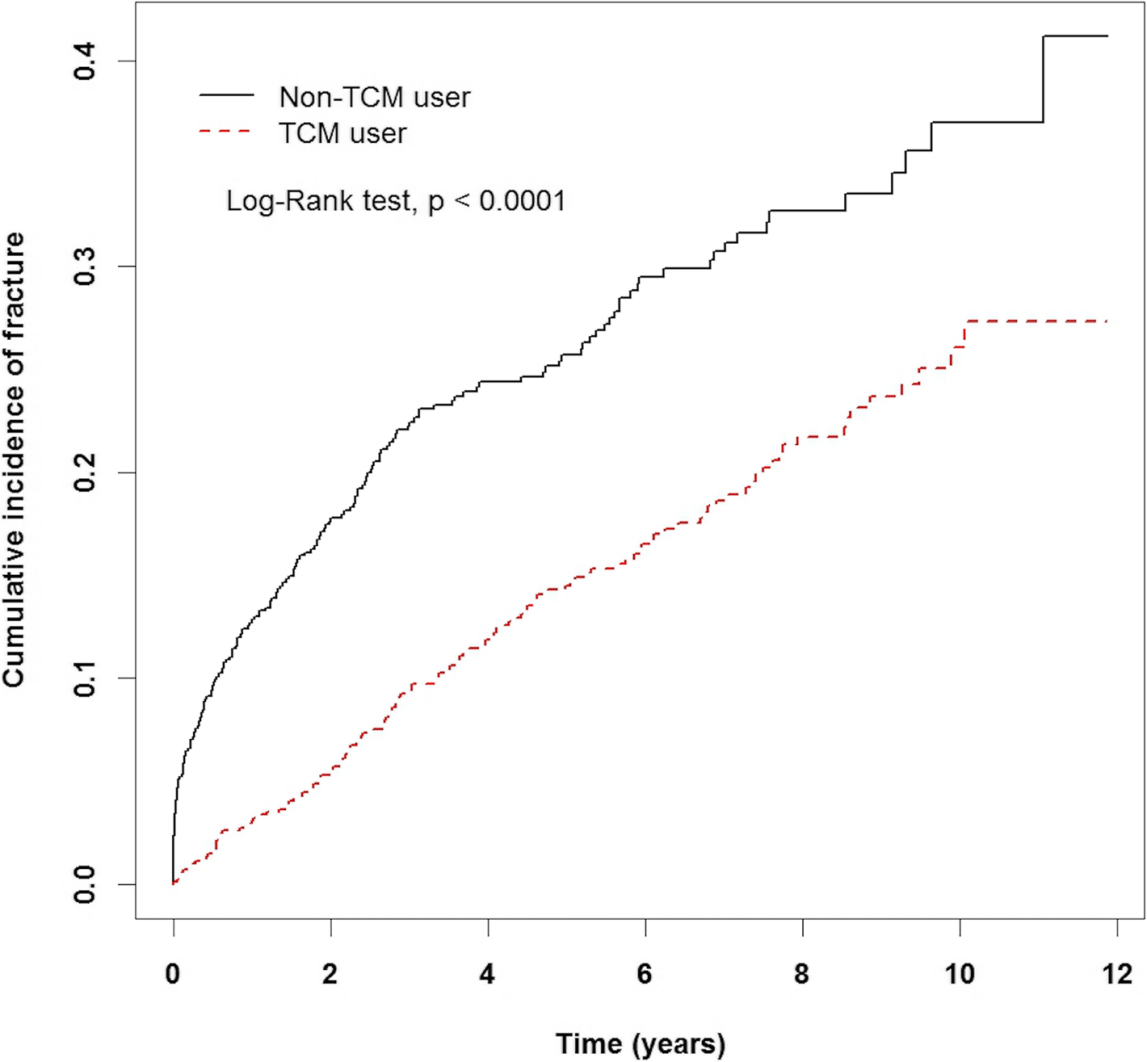
Kaplan–Meier curve of the difference between the TCM user and non-TCM user groups in the development of fracture

The Kaplan-Meier curves showed that osteoporosis patients using TCM had a lower incidence rate of fracture events than those not using it (*p* < 0.0001; Fig. 2).

Table 3 shows the distribution of TCM users according to their accumulated days of herbal use. Patients with < 30 days of Chinese herb medicine use per year (including non-TCM users) were selected as the reference. Patients who accumulated between 30 and 180 days of herbal use showed an aHR of 0.60 (95% CI: 0.43–0.84), and those with more than 180 days of herbal use showed an aHR of 0.37 (0.20–0.68). When analyzing patients with more than 30 accumulated days of herbal use, those who cumulatively used herbal prescriptions for more than 180 days had a lower risk of fracture (aHR: 0.63, 95% CI: 0.32–1.24) than in the compared cohort; however, this was not statistically significant.

**Table 3:** Hazard Ratios and 95% confidence intervals of fracture risk associated with cumulative use day of traditional Chinese herb medicine among osteoporosis patients

TCM used (days per year)	N	No. of Event	Hazard Ratio (95% CI)		Hazard Ratio (95% CI)	
			Crude	Adjusted^†^	Crude	Adjusted^†^
Non-TCM users or Chinese herb users < 30 days per year	1245	269	1(reference)	1(reference)	–	–
Chinese herb users (≥ 30 days per year) ^‡^						
30–180 days per year	270	43	0.63 (0.46–0.87)**	0.60 (0.43–0.84)**	1(reference)	1(reference)
180 days per year	93	11	0.42 (0.23–0.78)**	0.37 (0.20–0.68)**	0.65 (0.33–1.25)	0.63 (0.32–1.24)

Crude HR^*^ represented relative hazard ratio; Adjusted HR^†^ represented adjusted hazard ratio: mutually adjusted for age, gender, baseline comorbidity, and urbanization level in Cox proportional hazard regression

*p < 0.05, **p < 0.01, ***p < 0.001

The HR of the 10-single herb and multiherb products most commonly prescribed for the treatment of osteoporosis are listed in Table 4. Frequency meant how many times the single herb or multiple herb formula was used during the research. Number of person-days meant how many days the single herb or multiple herb formula was used during the research.

**Table 4:** Ten most common herbal formulas prescribed

Herbal formula	Frequency	Number of person-days	Average daily dose	Average duration for prescription
			(g)	(days)
Single Herb				
Eucommiae Cortex (Du-Zhong)	803	10,832	1.2	13.5
Salviaemiltiorrhizae Radix (Dan-shen)	592	7850	1.3	13.3
Chaenomelis Fructus (Mu-gua)	520	6651	1.0	12.8
Achuranthes (Huai-niu-xi)	432	5181	1.1	12.0
Dipsaci Radix (Xu-Duan)	393	4701	1.3	12.0
Sepiae Endoconcha (Haipiaoxiao)	337	4476	1.4	13.3
Corydalis Rhizoma (Yan-hu-suo)	346	4284	1.5	12.4
Spatholobi Caulis	272	3570	1.4	13.1
Testudinis Plastrum (Gui-ban)	279	3477	0.9	12.5
Drynariae Rhizoma (Gu sui-bu)	259	3282	1.3	12.7
Multiple Herb Formula				
Du Huo Ji Sheng Tang	1109	13,795	5.7	12.4
Gui Lu Er Xian Jiao	433	8083	7.0	18.7
Shu Jing Huo Xue Tang	564	7427	4.6	13.2
zuo Gui Wan	516	5625	5.2	10.9
ji Sheng Shen Qi Wan	308	4478	5.1	14.5
Zhi Bai Di Huang Yin	322	4365	4.7	13.6
Hu Qian Wan Without Hugu	312	4211	7.2	13.5
You Gui Wan	302	3982	4.3	13.2
Ma Zi Ren Wan	219	3572	1.8	16.3
Xiang Sha Liu Jun Zi Tang	220	3423	4.2	15.6

## Discussion

With the increase in osteoporosis prevalence and incidence, prevention of osteoporotic fracture is of great importance [14]. People have become more interested in natural products in recent years and TCM is becoming a common choice in complementary and alternative medicine. There are some difficulties when surveying the preventive effect of TCM for osteoporotic fracture in operational studies. First, a long follow-up time is required for bone mass and fracture events. The longest reported follow-up time in Western medicine for osteoporosis was ten years [15]. Therefore, most studies focus on the benefit to bone health [16] rather than the fracture rate. Secondly, a common problem of studying TCM is that it is difficult to quantify Chinese herbs. Current Chinese herb studies focus on extracts or simple herbs [9, 17, 18], which greatly differ from those used in the clinical setting. For the above reasons, there are no studies on the fracture incidence following TCM therapy in patients with osteoporosis. Therefore, we could conduct this survey using NHIRD analysis.

Our results showed a decreased risk of fracture following the use of TCM therapy among osteoporosis patients (HR: 0.47, 95% CI: 0.37–0.59; *p* < 0.0001). The follow-up period was also longer in the TCM user group than in the non-TCM user group (5.38 and 3.75 years, respectively). It means TCM use might delayed the occurrence of fracture after osteoporosis was diagnosed. The Kaplan–Meier curve also demonstrated that patients who took TCM had a lower incidence of fracture. In addition, our study demonstrated that the longer the use of TCM, the lesser the fracture rate. Patients who took TCM between 30 to 180 days were at less risk than those who took TCM for less than 30 days. Similarly, patients who took TCM for more than 180 days were at less risk than those who took TCM between 30 to 180 days. This result strengthens the relationship between TCM and osteoporotic fracture.

We should consider the possibility of a decreased fracture rate after using TCM therapy. First, TCM may improve bone strength [16], while falls are also a prominent factor for which one tenth lead to fracture [19]. Some studies demonstrate that the most important cause of a fracture is a fall rather than bone strength [20–22]. TCM may improve the quality of life by reducing limb pain or strengthening muscle endurance [23–25]. Some Chinese herbal clinical trials conducted in China showed that people who took Chinese herbs had a better quality of life or reduced symptoms including pain, muscle fatigue, and limited mobility. While these trials were included in a review study [26], they did not match the standard of peer-reviewed journals.

The frequency of major osteoporotic fractures varies in different races, especially in hip fractures, with rates varying by > 200-fold. White women have a higher fracture risk than black women. Furthermore, variation was also observed in different regions with northern Europe and Mediterranean areas experience the highest rates and the lowest rates, respectively [27]. Based on these differences, it is difficult to conclude the benefit of TCM when comparing ethnic groups.

The most commonly prescribed single herbs and formulas were presented in Table 4. While this is not the discussion point of our research, it may be important for clinicians and researchers. The results provide future research candidates for basic and clinical trials. Some herbs have been proven to be beneficial to bone health, such as Eucommiae Cortex (Du-Zhong) [28], Achuranthes (Huai-niu-xi) [29, 30], Salviae miltiorrhizae Radix [18], Dipsaci Radix (Xu-Duan) [31], Testudinis Plastrum (Gui-ban) [32–34], Drynariae Rhizoma (Gu sui-bu) [35–37], Du Huo Ji Sheng Tang [38], and zuo Gui Wan [39]. We found patients lived in highly urbanized areas were more likely to receive TCM treatment. In addition, some comorbidities showed significant difference between two groups in baseline. It might mean the patients with chronic obstructive pulmonary disease and hyperlipidemia preferred to receive TCM, and the patients with cancer were not disposed to use TCM. Besides, less cancer rate might also mean patients in TCM group take care themselves better than another group usually. Because of this, they go further to seek TCM treatment when osteoporosis is diagnosed.

There are some limitations to our research. Firstly, we were unable to include medicines taken at the patient’s own expense. According to the specification of the NHI program, Western medicine for osteoporosis can only be applied after a fracture occurred. It is possible that the patients source such medicines at their own expense when they were diagnosed with osteoporosis before a fracture happens. Secondly, some data related to fractures, such as a patient’s exercise, lifestyle, BMI, alcohol, and cigarette use is not available from the NHI program. Thirdly, the Kaplan–Meier curve might be influenced by economic levels and patient severity. However, we can conclude that TCM might have a positive impact on the prevention of osteoporotic fracture from Tables 2 and 3. Moreover, this study is derived from a very large, well-indicated data set, which provided a practical method to investigate the effect of TCM in osteoporotic patients. Our study not only reveals the preventative value of TCM use for patients with osteoporosis in the clinical setting, but also provides valuable information regarding the most common prescriptions provided to osteoporotic patients.

## Conclusions

In conclusion, our study had a relatively large population and long follow-up time, which demonstrated that TCM might have a positive impact on the prevention of osteoporotic fracture. Further research is needed to verify the causal relationship between TCM and the outcomes. More clinical trials are also required to confirm whether this relationship is true in non-Asian patients.

## Abbreviations

HR: Hazard ratios
LHID2000: Longitudinal Health Insurance Database 2000
NHI: National Health Insurance
NHIRD: National Health Insurance Research Database
PTH: Parathyroid hormone
TCM: Traditional Chinese medicine
